# Future Prospects and Challenges in Mucormycosis Research

**DOI:** 10.3390/jof11080545

**Published:** 2025-07-22

**Authors:** Georgios Chamilos, Ulrike Binder, Victoriano Garre

**Affiliations:** 1 Laboratory of Clinical Microbiology and Microbial Pathogenesis, School of Medicine, University of Crete, 71110 Heraklion, Greece; 2 Institute of Molecular Biology and Biotechnology, Foundation for Research and Technology, 71300 Heraklion, Greece; 3 Institute of Hygiene and Medical Microbiology, Medical University Innsbruck, Schöpfstrasse 41, 6020 Innsbruck, Austria; ulrike.binder@i-med.ac.at; 4 Departamento de Genética y Microbiología, Facultad de Biología, Universidad de Murcia, 30100 Murcia, Spain; vgarre@um.es

Mucormycosis is an increasingly important, life-threatening human fungal infection caused by Mucorales, and it has limited therapeutic options, a lack of biomarkers for early diagnosis, and incompletely characterized immunopathogenesis. Mucorales possess unique virulence factors and epigenetic mechanisms that control fungal growth, pathogenicity, and resistance to antifungal agents. Furthermore, mucormycosis has distinct clinical and epidemiological features, which reflect specific underlying mechanisms of immune dysfunction. Specifically, the ability of Mucorales to induce necrotizing, angioinvasive disease in patients with certain metabolic abnormalities implies that host metabolites play a significant regulatory role in fungal pathogenicity. Furthermore, it is possible that uncharacterized, evolutionarily conserved metabolic host defense mechanisms inhibit this class of fungal pathogens. Progress in understanding the immunobiology of mucormycosis remains constrained by (a) the scarcity of human biological samples collected, (b) the lack of broadly available genetic tools and strains engineered to dissect host–Mucorales interplay, (c) the variety of fungal species that cause the disease, and (d) insufficient funding resources. Collaborative and coordinated efforts between experts in fungal biology, genetics and genomics, immunology, pathogenesis, and clinical research on mucormycosis are needed to overcome these challenges. These synergistic interactions would lead to new funding opportunities and yield novel insights into disease mechanisms, ultimately enabling the development of innovative therapies targeting Mucorales’ pathogenicity and improving clinical outcomes for this disease.

Mucorales comprise a group of ubiquitous filamentous fungi (molds) that are prevalent pathogens in plants [1] and animals [2,3], including invertebrate hosts [4,5,6]. In humans, Mucorales cause mucormycosis, a life-threatening, necrotizing, angioinvasive infection with incompletely understood pathogenesis and limited therapeutic options [4,7,8,9,10,11,12,13,14,15]. Globally, *Rhizopus* spp. are the predominant causative agents of mucormycosis [7,16]. Mucormycosis is associated with an unacceptably high mortality rate of 50%, exceeding 90% upon dissemination [7]. Its poor outcomes are largely attributed to limited therapeutic options due to the intrinsic resistance of Mucorales to most antifungal drugs, the underlying immune dysfunction, and extensive tissue necrosis, which impedes the effective delivery of antifungal agents to the site of infection and necessitates radical surgery. Over the past two decades, significant advances have been made in understanding the epidemiology, pathogenesis, and treatment of mucormycosis. D.P. Kontoyiannis’ research group has played an instrumental role in coordinating many of these efforts. However, significant gaps remain in our understanding of the immunobiology of mucormycosis. Elucidating the early immunopathogenic mechanisms of mucormycosis is essential for the design of effective antifungal drugs and innovative prophylactic and therapeutic interventions, ultimately aiming to improve the disease’s clinical outcomes. 

Similarly to other invasive mold infections (IMIs), mucormycosis affects severely immunocompromised patients with defects in terms of the number and/or function of phagocytes, including patients with a hematological malignancy and chemotherapy-induced myelosuppression or aplastic anemia, alongside transplant recipients (Table 1) [4,17]. However, unlike other IMIs, mucormycosis predominantly affects an ever-expanding group of patients with unique metabolic abnormalities, including poorly controlled diabetes mellitus (DM), diabetic ketoacidosis or other types of acidosis, acquired iron overload syndromes, deferoxamine treatment, malnutrition, severe trauma, and burns [4,7]. Furthermore, incompletely characterized immunometabolic abnormalities induced by COVID-19 in the setting of corticosteroid therapy and DM resulted in an epidemic of mucormycosis in India and other parts of the world [18]. Altogether, these findings suggest that unidentified host metabolic defense mechanisms prevent Mucorales infections in healthy individuals.

The ability of Mucorales to evade host immune responses and induce angioinvasive, necrotizing disease—particularly in patients with metabolic abnormalities—suggests that host metabolic factors play a crucial role in modulating fungal growth and virulence. In fact, several lines of evidence suggest that mucormycosis is a metabolic disease. Regarding pathogens, iron assimilation pathways [19] and specialized mechanisms that promote xenosiderophore acquisition [20] are crucial virulence factors of Mucorales. Excessive iron availability, in the context of iron overload or acidosis [21], hyperglycemia, and a high number of ketone bodies, promotes angioinvasion through the expression of epithelial and endothelial receptors for Mucorales CotH invasions [22]. Additionally, β-hydroxy butyrate (BHB) triggers fungal growth via uncharacterized mechanisms [21]. Interestingly, *R. delemar* produces novel peptides that alter iron homeostasis and promote intracellular growth in macrophages [23]. 

From the host perspective, nutritional immunity via iron limitation inhibits the intracellular growth of Mucorales inside macrophages [24]. However, swollen *R. delemar* spores evade phagocytosis and induce fulminant disease and rapid death following pulmonary infection in immunocompetent mice. The release of the potent mycotoxin mucoricin during the germination of Mucorales largely accounts for their ability to induce significant tissue necrosis and promote fungal invasion [25]. Therefore, the inhibition of fungal growth both intracellularly and in the extracellular space plays a crucial role in the host’s defense against Mucorales. These hypothetical metabolic host defense mechanisms could specifically target Mucorales growth inside the host. In this context, understanding the pathways that regulate the transition from saprophytic to pathogenic metabolism and the growth of Mucorales could shed light on the specialized host effector mechanisms that target this class of pathogens. Achieving this goal requires increasing the availability of genetic engineering tools and fungal models, which are currently limited to only the species *Mucor* and *Rhizopus*. Studies on *Mucor* spp. have demonstrated the crucial role of mitochondrial metabolism on fungal growth and virulence via the downregulation of PKA signaling [26,27]. Notably, a unique epigenetic pathway transcriptionally regulates the growth of Mucorales in vitro and in vivo through the symmetric N-6 adenine methylation (6 mA) of DNA [28,29]. Interestingly, mitochondrial metabolites and enzymes directly regulate chromatin-modifying enzymes to control the growth of cancer cells [30]. Therefore, investigating metabolic signals that modulate the epigenetic mechanism regulating fungal growth is crucial. Furthermore, characterizing the transcriptional networks that link growth regulation to the pathogenesis of Mucorales will be essential for our molecular understanding of the immunobiology of this infection. 

Apart from the effects of host metabolic factors on fungal pathogenicity, these molecules also have a direct impact on the immune system. For example, excessive amounts of BHB inhibit ROS production and impair the antifungal effector properties of neutrophils [21]. Additionally, metabolic abnormalities predisposing to mucormycosis, including iron overload, systemic acidosis, malnutrition, and poorly controlled diabetes, have complex and pleotropic effects on the systemic immune response. Nonetheless, the physiological immune responses against Mucorales remain incompletely characterized at the molecular level. Specifically, although alveolar macrophages (AMs) have a non-redundant role in host defense against Mucorales [24], the immunometabolic responses and effector mechanisms that eliminate fungal spores following phagocytosis currently remain unknown. Similarly, although neutropenia is a predominant risk factor for mucormycosis [4,7], neutrophil immune responses against Mucorales in health and disease have not yet been explored. Furthermore, the role of adaptive immunity in physiological host defense against mucormycosis has not been evaluated (Table 2). 

The timely initiation of appropriate amphotericin B-based therapy markedly improves outcomes in patients with mucormycosis [31]. However, early diagnosis is challenging because of the lack of reliable surrogate biomarkers and the clinical similarities between pulmonary mucormycosis and other invasive mold infections (IMIs) [32]. Although the reverse halo sign on CT imaging [33,34], especially when combined with characteristic clinical and epidemiological features, can demonstrate an increased likelihood of mucormycosis [35], specific host and pathogen biomarkers are required to improve diagnosis of the disease. The recent implementation of commercially available serum Mucorales quantitative PCR (qPCR) for high-risk hematological patients represents an important diagnostic tool [36]. Nonetheless, the performance of qPCR is suboptimal in mucormycosis patients with diabetes mellitus as the main underlying risk factor [36]. A more in-depth molecular understanding of Mucorales biology will facilitate the development of novel diagnostic assays. 

Overall, a systems medicine approach is needed to comprehensively characterize the dynamic interplay between Mucorales and the immune system, which will facilitate the identification of targeted therapeutic strategies that can disrupt fungal pathogenicity at the early stages of the disease (Figure 1). To achieve this, it is important to systematically explore the effects of metabolic disorders and certain metabolites in innate host defense pathways that confer protective immunity against Mucorales using relevant animal models of mucormycosis. The use of alternative mini-host models that allow for high-throughput experimental approaches to testing candidate novel compounds and genetic screening could also provide invaluable information on the evolutionary mechanisms of host–Mucorales interactions [5]. For examples, the role of serum transferrin in systemic nutritional immunity against *R. delemar* is evolutionary conserved from *Drosophila melanogaster* to humans [37]. 

Advancing our molecular understanding of mucormycosis’ immunopathogenesis is hindered by significant challenges. These include (a) the scarcity of clinical samples and human biorepositories available to validate findings from preclinical models, (b) the limited availability of genetic tools and engineered fungal strains to study the dynamics of host–fungal interplay, and (c) the lack of established animal models for studying emerging groups of patients (e.g., CAM) and the different aspects of the disease. To overcome these challenges, there is a pressing need for collaborative, interdisciplinary research initiatives. Fostering synergy among experts in fungal biology, genomics, immunology, pathogenesis, and clinical care will create an ideal environment for groundbreaking discoveries in fungal biology and immunology. The establishment of these effective collaborative networks will ultimately depend on adequate funding support from national and international agencies, in alignment with the recommendations of the World Health Organization in the Fungal Priority Pathogens List, which includes Mucorales. Advancing our molecular understanding of the immunobiology of mucormycosis will transform novel discoveries into better diagnostic, therapeutic, and preventive strategies to ultimately improve disease outcomes. 

**Table 1:** Unique host factors associated with susceptibility to Mucorales in comparison with *Aspergillus fumigatus* and other molds.

Predominant IMI Due to *A. fumigatus* and Other Molds [7,38,39]	Shared Risk Factors for IMI [18,40,41]	Unique Risk Factor for Mucormycosis [4,38]
**Primary immunodeficiency** Genetic defects in NADPH oxidase complex (chronic granulomatous disease; CGD) * CADR9 deficiency	**Defects in number or function of phagocytes in** hematological malignancy and transplant recipients Prolonged, persistent neutropenia High doses of corticosteroids Aplastic anemia	**Primary immunodeficiency** Acquired STAT1 function [42] Papillon–Lefevre syndrome [43]
**Acquired immune defects** Influenza-associated aspergillosis (IAPA) #	**Immune deactivation induced by bacterial sepsis**. **Severe fever with thrombocytopenia syndrome** (SFTS). **Immunosuppression related to ibrutinib and other small-molecule kinase inhibitors** (SMKIs). **COVID-19-associated** aspergillosis (CAPA) and COVID-19-associated mucormycosis (CAM).	**Metabolic disorders** Poorly controlled diabetes mellitus (DM) Diabetic ketoacidosis (DKA) Other forms of acidosis Acquired iron overload; Deferoxamine therapy Malnutrition **Trauma and burns**

* Mucormycosis almost exclusively occurs in CGD patients receiving corticosteroids [44]. # Mucormycosis following influenza has been reported in patients receiving steroids or with underlying risk factors (e.g., neutropenia, poorly controlled DM) [45].

**Table 2:** Important questions in immunopathogenesis of mucormycosis.

What is the physiological role of adaptive immunity in host defense against Mucorales?
What is the role of adaptive immunity in inflammatory immunopathology associated with sepsis-induced mucormycosis?
Do professional phagocytic cells employ distinct mechanisms to eliminate inhaled Mucorales spores compared to other fungal pathogens?
Is there a role of host metabolism in immunity against Mucorales?
Are there distinct metabolic pathways regulating Mucorales growth and virulence as compared to other fungal pathogens?
What is the role of host metabolites in fungal growth and virulence?
What is the effect of certain host metabolites on physiological immune responses against Mucorales
What is the effect of certain metabolic abnormalities, including iron overload, acidosis, and malnutrition, on the function of the immune system?
Which immunometabolic alterations are related to COVID-19-associated mucormycosis (CAM)?
What is the impact of genetic defects on the development of mucormycosis?

## Figures and Tables

**Figure 1:**
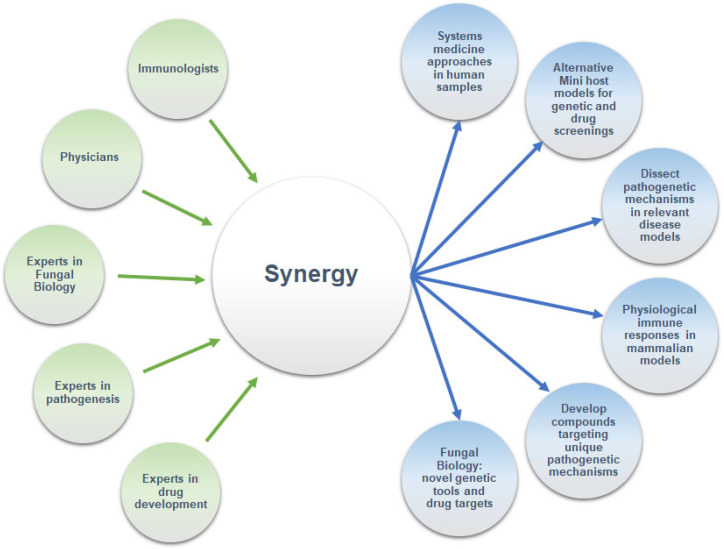
A roadmap for synergistic research interactions toward novel drug discoveries and enhanced management of mucormycosis.
